# Supplementary material for: Patterns of Intron Gain and Loss in Fungi
Source: PLoS Biol. 2004 Nov 30;2(12):e422. doi: 10.1371/journal.pbio.0020422 (PMC532390; doi:10.1371/journal.pbio.0020422)
Supplement: Table S1 — Also available at http://genes.mit.edu/NielsenEtAl/. (4.3 MB ZIP). [file pbio.0020422.st001.zip › NielsenEtAl/html/325.html]

AN2907.1.NCU05889.1.MG03317.1.FG06073.1


```
 CLUSTAL W (1.82) Multiple Sequence Alignments - Introns Inserted


Sequence 1: NCU05889.1	443 aa
Sequence 2: MG03317.1	446 aa
Sequence 3: FG06073.1	439 aa
Sequence 4: AN2907.1	448 aa
Alignment Length: 461 aa
Number Identitical Residues: 212 aa
Alignment Score (without introns) 10834


MG03317.1 	----MAPSAESEDN----------SLAQFDLLPKLVKNLDRHLIFPLLDAASAQYYDEET
NCU05889.1	----MASTSPATTNG-------DVAPANYDLVLKLAPHLDRHMIFPLLEFNAGRLKEDET
FG06073.1 	----MEDVLNAVSAD------------GSSIMPRLAPNLSRHLLFPLIQFESERAEEQGL
AN2907.1  	MAANVPPSAEALTSGGAAHKTAEDIANQYDLLPKLIPYLDRHLVFPLLEFSSGQ------
          	 ::.:     :   ..:: .::.      .:: :*   *.**::***::  : :      

MG03317.1 	NEPRDVDKAREMTKAKFELLKKTNMTDYVANLHCELEGTDEPPAEYAEKRQKVIKQLQDF
NCU05889.1	------DKAREILAAKYALLKKTNMTDYVANLYCELEGLKEPPAEYAERRQKVFHQLEKY
FG06073.1 	D-----AKAKELLSGKIKLLEDTNMTDYVATLYCELHGVENAPEEYNKKRQEVLSQLENY
AN2907.1  	------DDDSEIVRAKYELLKHTNMTDYVANLWQEINNSDTIPDEFVKKREEVLAKLQHY
          	       .  *:  .*  **:.********.*  *:.. .  * *: ::*::*: :*:.:

MG03317.1 	EDSVSKLRDLLDREEVISNLRSDKVANLEFLKKEHD0VTIEMVTALYDFGNFQYSCGNYP
NCU05889.1	EQETAKITELLQRDDVVNNLRSDKVANLEFLKKEHD0VTIDMVNALYDFGQLQYSCGNYA
FG06073.1 	EQATAKIADLLTQDEVVNGLRSDKVANLEFLKNQHG0VTMEMVNALYDFGQFQFRCGQYG
AN2907.1  	QNESEKITELLQDEAVVGNLRSDKVANLRFLEEQHG~VTIEMVNSLYDYGRFQYSCGSYG
          	::   *: :**  : *:..*********.**:::*. **::**.:***:*.:*: **.* 

MG03317.1 	AAAELLYQFRVLSTDNDKVSAATWGKLASEILSGNWEGAMEELKKVREDIDSRLFNNPLA
NCU05889.1	DASELLYRFRVLSTDNDKVSYATWGRLACEILTMNWESAMEELQKVRESIDTRLANNPLA
FG06073.1 	PAADMLYQFRVLSTDNDKVSAATWGKLASEILQTNWESAVDELKNVRENIDSKLFNNPRA
AN2907.1  	NAAELLYQFRVLSTDNDKVASATWGKLASEILTTNWEAAMEEVQKAKDSIETRLFNNPVG
          	 *:::**:***********: ****:**.***  ***.*::*:::.::.*:::* *** .

MG03317.1 	QLQHRTWLTHWALFPLFNDENSREALLEMFFSPNYINTIQTSCPWILRYLAVVVIAGRGR
NCU05889.1	QLQHRTELVHWALFPLFNYDKAREPLLDLFFNAGFINTIQANSPWILRYLTVAVITNRGR
FG06073.1 	QLDHRTMLLHWSLFPLFNWEGAREPILEMFFSAPFINTIQTSCPWILRYLIAAVITGRGR
AN2907.1  	QLHNRSWLIHWSLFPFFNHDPARDVLTDLFFSPAYINTIQTSCPWILRYLAAAVITNRNR
          	**.:*: * **:***:** : :*: : ::**.. :*****:..******* ..**:.*.*

MG03317.1 	AR-NTGAYQKQLKDVVRIVKQEGYEYSDPVTDFVRALYIDFDFEEAQRQLPRAEEVLRTD
NCU05889.1	AK-NAGVHQKQMKDVVRIVKQEAYEYQDPITRFVHALCIDFDFEEAQQQLVLAEEVLRSD
FG06073.1 	AR-NSSIQQKQIKDIIRYVRQEAYEYTDPITQFVNALYIAHDFEAAREALSMAAEVCRSD
AN2907.1  	AHKNSSVYQKQLKDLIRVVRQEGYEYSDPITDFVKALYVDFDFEEAQKKLGEAEEVLRGD
          	*:.*:.  ***:**::* *:**.*** **:* **.** : .*** *:. *  * ** * *

MG03317.1 	FFLMSIGDSFVDAARHLFFESYCKIHARIDLK~RLSEQLGLNVDEGEKWIVNLIRDTRLD
NCU05889.1	FFLLAHADDFVDSARHLIFESYCKIHARISLK~DLSARLGLNNDDAEKWIVNLIRDTRLD
FG06073.1 	FFLASSADAFVDAARHLICESYCKIFSRMNIR~DLSAKLGLNPDDGEKWIVNLIRETRLD
AN2907.1  	FFLVSAADAFVEAARHLISESYCKIHQRIDIK2DLSTRLGLNQDEGEKWIVNLIRDTRVD
          	*** : .* **::****: ******. *:.::  ** :**** *:.*********:**:*

MG03317.1 	AKIDFQEGTVVMNHPNSSVYQQVIERTKGGFFRTQVLSAAVAR
NCU05889.1	AKIDYKEGTVVMNHPPSSVYQQVIERTKGGFFRTQVLTAAVAR
FG06073.1 	AKIDSQDGTVIMNHPPNNVYQQVIEKTKGGFFRTQVLNAAVSK
AN2907.1  	AKIDYKEGTVIMNHPPQSVYQQVIEKTKGAFFRTQVLRFVAS-
          	**** ::***:**** ..*******:***.*******  ..:
```
